# Zen and deep evolution: The optical delusion of separation

**DOI:** 10.1111/eva.12620

**Published:** 2018-03-23

**Authors:** Fred W. Allendorf

**Affiliations:** ^1^ Division of Biological Sciences University of Montana Missoula MT USA

**Keywords:** extinction, interdependence, karma, mindfulness, religion, science

## Abstract

The Buddha taught that everything is connected and constantly changing. These fundamental observations of the world are shared by ecology and evolution. We are living in a time of unprecedented rates of extinction. Science provides us with the information that we need to address this extinction crisis. However, the problems underlying extinction generally do not result from a lack of scientific understanding, but they rather result from an unwillingness to take the needed action. I present mindfulness and meditative aspects of Zen practice that provide the deeper “knowing,” or awareness that we need to inspire action on these problems.

The extinction of species, each one a pilgrim of four billion years of evolution, is an irreversible loss. The ending of the lines of so many creatures with whom we have traveled this far is an occasion of profound sorrow and grief. Death can be accepted and to some degree transformed. But the loss of lineages and all their future young is not something to accept. It must be rigorously and intelligently resisted.

Gary Snyder (1990)

## 1 INTRODUCTION

The Buddha taught that everything is connected and that everything is constantly changing. These fundamental observations of the world are shared by ecology and evolution. Ecology teaches that everything is connected through both biotic and abiotic interactions. We know from evolution that everything is connected by genealogy and that everything is constantly changing. We are facing an extinction crisis. Ecology and evolution provide us with the scientific background that we need to address these problems. However, the environmental problems underlying extinction generally do not result from a lack of scientific understanding but rather result from an unwillingness to take the needed action. Zen provides the deeper “knowing,” or awareness, that we need to motivate action on these problems.

Buddhism is a major global religion with many complex systems of differing beliefs. In this paper, I have used Rahula (1974) as my primary source for the teachings of the Buddha because he presents the original teachings, rather than the interpretations by any of the major schools of Buddhism. I also have relied considerably on the writings of Tenzin Gyatso, the current Dalai Lama, because of his interest in the relationship between science and Buddhism (e.g., Dalai Lama, 2005). Finally, I emphasize the Zen teachings of Thich Nhat Hanh (1998), which are based on the Buddha's teachings of mindfulness, awareness, and meditation to live in the present moment (Rahula, 1974: page 72). Other major religions have contemplative traditions that are similar to the practice of Zen in Buddhism (e.g., Sufism in Islam). In the rest of this paper, I generally use the term Buddhism to reflect the teachings of the Buddha as presented by Rahula (1974).

My overall objective in this paper is to unite the sciences of ecology and evolution with the spiritual practice of Zen in order to inspire actions to address the extinction crisis that we are currently facing. I do this by addressing the following three points:

1. Zen and science are both based upon empirical observations of the world.
2. Zen and science both tell us that there is no separation between humans and the world around us.
3. Ecology and evolution provide the scientific background needed to address the biodiversity crisis; Zen provides the deeper knowing that will motivate our action to address this problem.

## 2 ZEN AND SCIENCE

Spirituality and science are different but complementary investigative approaches with the same greater goal, of seeking the truth.

Dalai Lama (2005: page 4)

### 2.1 Both science and Zen are based on empirically verified observations

Science is based on making observations of the world, forming hypotheses which explain those observations, and finally testing those hypotheses with empirical data. The Buddha used this method when questioned by people in the village of Kalama why his teachings should be followed rather than the teachings of other spiritual teachers who visited the village. In the Kalama Sutra, the Buddha said that his teachings should be accepted only if they agree with reason, common sense, and are found to lead to “benefit and happiness” after they are experienced (Rahula, 1974: page 2).

Some have argued that science and religion (including Buddhism) are inherently incompatible. As an evolutionary biologist, I am well aware of the conflicts between science and religion. Many people in the United States reject the scientific findings of evolution and believe that God created humans in their present form sometime within the last 10,000 years or so. Such conflicts result from placing religious doctrines above experience and reason. However, Buddhism is based upon the teachings of the historical Buddha who maintained that his understanding came only from his own experience, not from any god or external source (Rahula, 1974: page 1). In the words of the Dalai Lama (2005: page 3), “if scientific analysis were conclusively to demonstrate certain claims in Buddhism to be false, then we must accept the findings of science and abandon those claims.”

Coyne (2015: page 306) presented several reasons why Buddhism is incompatible with the “primacy of fact”. One is that the Dalai Lama and other Buddhist writers have said that Buddhism cannot accept the randomness of mutations. I address this issue in the next section. Coyne also states that Buddhism accepts two supernatural claims: karma and reincarnation. However, karma is not a supernatural phenomenon in the way that it is often perceived in the Western world. According to Rahula (1974: page 32), “The theory of karma is the theory of cause and effect, of action and reaction; it is a natural law, which has nothing to do with the idea of justice or reward and punishment.”

The concept of reincarnation is more complicated. Many Buddhists (e.g., Tibetan) accept the notion of reincarnation and past lives. However, the Buddha taught that there are no such things as a permanent Self or Soul which can be reincarnated or reborn after death (Rahula, 1974: page 3). The Zen master Thich Nhat Hanh believes there is no reincarnation in Buddhism (Miller, 2012). This is how he explains the continuation of life after the death of the body: “But when you see me in my speech and my actions, you see that they continue me. When you look at my disciples, my students, my books, and my friends, you see my continuation. I will never die. There is a dissolution of this body, but that does not mean my death. I continue, always.”

### 2.2 Randomness and evolution

As pointed out by Coyne (2015), some Buddhists believe that the belief in karma and causation rule out the role of chance in evolution. “From the Buddhist's perspective, the idea of these mutations being random events is deeply unsatisfying for a theory that purports to explain the origin of life.” (Dalai Lama, 2005: page 112). This view occurs in many Buddhist writings that reject the spontaneous appearance of random mutations because they are in conflict with the notion of cause and effect in karma (e.g., Lopez, 2010; Low, 2008).

The arguments that the randomness of mutations is incompatible with karma are simply wrong. They do not reflect either the teachings of the Buddha or the principles of evolution. There is nothing in the Buddha's teachings of cause and effect that rejects the role of chance. Genetic drift is one of the major factors that cause allele frequencies change in populations. Certainly, those who do not accept the randomness of mutations on the basis of karma would not reject the role of random changes caused by genetic drift in evolution because of karma. Moreover, genetic drift does follow the principle of cause and effect. Small population size will cause loss of genetic variation and widespread allele frequency changes. However, it is also random because we cannot predict which alleles will increase and which will decrease in frequency.

It is interesting to note that one of the most heated controversies among evolutionary biologists also revolved around the importance of randomness and genetic drift in evolution. Under the neutral theory of molecular evolution (Kimura, 1983), the majority of allelic molecular differences are selectively neutral so that changes in allele frequency are determined primarily by genetic drift, not natural selection. The opposing view was that virtually every allelic difference affects some aspect of the genotype and therefore is subject to the effects of natural selection (Clarke, 1979; Wills, 1973); Hey (1999) and Nei (2005) provide contrasting and complementary reviews of the history of this controversy.

## 3 EMPTINESS (NONSEPARATION IN SPACE)

A human being is a part of the whole, called by us “Universe”, a part limited in time and space. He experiences himself, his thoughts and feelings as something separated from the rest – a kind of optical delusion of his consciousness.

Albert Einstein (1950)

It is a basic tenet of ecology that species within an ecosystem are interconnected by a web of complex ecological interactions (Odum & Barrett, 2005). One of the first demonstrations of this was Forbes (1925) who presented a paper in 1887 that discussed the relationships among species of plants, animals, and microbes within a series of small lakes in the Midwest of the United States.

The Buddhist concept of interconnectedness or emptiness (all things are empty of a separate self) is represented by the metaphor of the Jewel Net of Indra: “Far away in the heavenly abode of the great god Indra, there is a wonderful net which has been hung by some cunning artificer in such a manner that it stretches out infinitely in all directions. In accordance with the extravagant tastes of deities, the artificer has hung a single glittering jewel in each “eye” of the net, and since the net itself is infinite in dimension, the jewels are infinite in number. There hang the jewels, glittering like stars in the first magnitude, a wonderful sight to behold. If we now arbitrarily select one of these jewels for inspection and look closely at it, we will discover that in its polished surface there are reflected all the other jewels in the net, infinite in number. Not only that, but each of the jewels reflected in this one jewel is also reflecting all the other jewels, so that there is an infinite reflecting process occurring” (Cook, 1977).

The ecologist David Barash (1973) discussed the parallels between Zen Buddhism and ecology. He felt that the interdependence and unity of all things was fundamental to both the practice of Zen and the science of ecology. In addition, both share a common nondualistic view of the fundamental identity of subject and surroundings. A bison cannot be understood in isolation from the prairie; understanding requires study of the bison‐prairie unit. He concluded that “the very study of ecology is the elaboration of Zen's nondualistic thinking.”

Through meditation and the cultivation of mindfulness, Zen acts to develop the realization that self and world are not separate. Thich Nhat Hanh offers the following guidance: “If we want to continue to enjoy our rivers ‐ to swim in them, walk beside them, even drink their water ‐ we have to adopt the non‐dual perspective. We have to meditate on being the rivers so that we can experience within ourselves the fears and hopes of the rivers. If we cannot feel the rivers, the mountains, the air, the animals, and other people from within their own perspective, the rivers will die and we will lose our chance for peace” (Nhat Hanh, 1991: page 105). Box 1 presents a guided meditation to help achieve this awareness of our physical connection to the world around us.

Box 1 **Guided meditation: The carbon cycle**

1 Imagine that you are tiny, incredibly tiny–the size of an atom. Imagine an atom, one of the two atoms of oxygen in one of the oxygen molecules that make up about 20% of the air in this room. As that atom is bumping around among the other gas molecules in the air of the room, suddenly it is sucked through your nostrils, and down into your lungs. Feel that fresh lungful of air that you have just breathed in, and imagine it from the oxygen atom's point of view.

This molecule that we breathed in from the air was released by a tree in a tropical rain forest in Malaysia. The tree took in carbon dioxide from the air and converted the carbon in that molecule into sugar, using the energy from the sun. Our oxygen molecule was then released as a waste product of that photosynthetic reaction. After expiration by the leaf, the molecule entered the atmosphere and traveled until it reached our nose, entered our body, where it was used to fuel our cellular metabolism.

Let's return our awareness to the oxygen atom within the cell. Suddenly, a commotion. The oxygen atom on which you ride is thrust into a chemical reaction, combining with a carbon atom that a nanosecond ago was bound to other carbons, hydrogens, and oxygens in a carbohydrate–a sugar molecule. That carbon, this morning, was part of the pancakes, or toast, or oatmeal, or whatever you ate for breakfast… You can feel the humming, the warm release of energy from that reaction… Energy that only this morning was “outside” of you, in your breakfast, now you! Feel it, deep in your muscle, or brain, or toe.

We just followed one oxygen molecule from a tree in Malaysia to our body back out into the atmosphere. There are trillions of oxygen molecules in every breath we take; every one of those molecules has a history of its own. Most are the product of photosynthesis in a plant; it could be from a tropical tree, from algae in the ocean, or grass on the plains of Africa. Every breath we take connects us directly to billions of plants throughout the world.

That carbon dioxide molecule, breathed out by you, bumping among leaves somewhere, finds its way into the inside of a leaf, through one of the leaf‐pores, the stomata, through which leaves “breathe.” In the leaf, the carbon dioxide is taken into cells, green cells full of the pigment chlorophyll. Bathed with light, again the jolting transformation of a reaction. The carbon atom, your partner on an atmospheric journey for so long, is suddenly pulled away, combining with water under the magical influence of light and chlorophyll into another sugar molecule, like the one it left from your breakfast that day… That carbon atom will become part of a wheat grain, honey from the nectar of a flower, a banana… To be eaten by another creature, powering its humming life when its energy is released from bondage by a reaction with oxygen. Maybe even to be eaten once again by you.

Our breath binds us to what is “outside” us: to the plants, who release the oxygen we need to take in; to the atmosphere, flowing restlessly forever around our blue planet; to sunlight energy, flowing down and trapped by the green miracle of a plant's leaf in the form of sugar, starch–food. In our breath, inside becomes out, and outside in; we are bound to the planet. Can you feel it–that interdependence–at the still point of your turning breath?

## 4 IMPERMANENCE (NONSEPARATION IN TIME)

It is a century now since Darwin gave us the first glimpse of the origin of species. We know now what was unknown to previous generations: that we are only fellow voyagers with other creatures in the odyssey of evolution. This new knowledge should have given us, by this time, a sense of kinship with other creatures; a wish to live and let live; a sense of wonder over the magnitude and duration of the biotic enterprise.

Aldo Leopold (1949)

Understanding evolution has led to the realization that we are kin to all other species on Earth. As pointed out by Leopold, this recognition of kinship should lead to an increased sense of caring for other species. Darwin (1837) also recognized this connection in the early stages of developing his concept of evolution: “If we choose to let conjecture run wild, then animals, our fellow brethren in pain, diseases, death, suffering and famine ‐ our slaves in the most laborious works, our companions in our amusements ‐ they may partake from our origin in one common ancestor ‐ we may be all netted together”. It is striking that Darwin used the same metaphor as the Jewel Net of Indra.

There is a traditional Zen koan which is often stated something like this: “What was your Original Face before your parents were born?” There is no such thing as a “correct” answer for a koan. This koan is sometimes interpreted as an invitation to contemplate one's ancestry. This inspired me to use the following guided meditation when I taught a class in Ecology and Buddhism (Box 2).

Box 2 **Guided meditation: When did your life begin?**

1

1. Go back to your birth, when convention says your life began. However, you existed in your mother's womb for many months before. The egg and sperm that joined to create your genome existed before they united. Your mother's egg divided to determine which genes she would pass to you while she was a fetus inside her mother, your grandmother. Contemplate your parents and your four grandparents. Who were they? How did they live?
2. Continue looking deeper. Your ancestors double each generation: two parents, four grandparents, eight great‐grandparents, and so on. Go back 1,000 years, some 40 generations. One thousand years ago, you had a trillion ancestors. The genes in every cell of your body were then shared among those ancestors spread around the globe: Europe, Asia, America, Africa. Who were they? How did they live?
3. One billion years ago. You have now been joined in your ancestral journey by all living species that we recognize in our daily lives. You are the wolf, the bear, the whale, the salmon, the pine tree. Your ancestors are simple single‐cell organisms living in the waters of the primitive Earth. Who were they? How did they live?
4. The last step in your journey: 4 billion years ago. There are no signs of life here. The stream of ancestors that you have been following has ended in a series of complex chemical reactions in which nonliving elements are becoming the simplest of possible living organisms. Contemplate the beginning of your life.

My own experience with this koan began in morning meditation next to a mountain lake on a backpack trip in Montana. I initially contemplated my parents and those few ancestors of whom I was aware. As an evolutionary geneticist, my mind traced the DNA that had been passed down from generation to generation since the beginning of human time. I seamlessly traced the DNA back to the ancestors of our species on the savannah of Africa, and beyond. The journey did not end until the beginning of life on Earth. Through continued deep contemplation of this koan, we can experience the concept of “nonself” in time by tracing our ancestry in evolutionary time. Looking deeply into this seemingly simple question, we can see that our life had no beginning, other than the origin of life itself on Earth. Tracing our ancestry such as in this guided meditation can also provide some genetic surprises about our relationships with our fellow humans (Box 3).

Box 3 **We are all related**

1

*Each of us is connected to the historical past in many ways – through the language we speak, the changed face of the earth around us, images, emotions, and preconceptions. The “ties of blood”, that is of genes and lineal descent, in some sense the most real of all connections, are also most mystical. Always hard to grasp imaginatively, they become still more elusive when a statistical element enters, when they connect us to millions of people, a part of the whole population that we can count but not identify. Our exercise of modeling numbers of ancestors opens the way to piquant speculation*.

Wachter (1980: page 92)

Consideration of our pedigrees reveals that we are all more closely related to each other than we generally appreciate. The numbers of our ancestors increase so quickly that it is soon unavoidable that we have common ancestors. The guided meditation in Box 2 led us through a journey back through our ancestors. Our number of ancestors doubles each generation: two parents, four grandparents, eight great‐grandparents, and so on. Going back *k* generations, we have 2^k^ ancestors. Let's assume that the mean human generation interval has been 25 years. One thousand years ago, we each had 1,099,511,627,776 ancestors.

My ancestry is from Europe. There were some 50 million humans alive in Europe one thousand years ago (Livi‐Bacci, 2017). At that time, I had over 20,000 times more ancestors in my pedigree than people in the population. Therefore, many people occupy numerous positions in my pedigree. This has been called “pedigree collapse” (Cann, 1988). How many of those 50 million people were my ancestors? We can estimate this if we make some simplifying assumptions (Derrida, Manrubia, & Zanette, 2000). It turns out that approximately 80% of the people alive at that time who reproduced are ancestors of all descendants of European ancestry today, and, 20% are ancestors of no living people. Thus, each of us alive today with European ancestry has exactly the same set of genealogical ancestors. Think of it, 8 out of 10 people who reproduced in northern Europe 1,000 years ago are the ancestors of all living people with some European ancestry.

All of us with some European ancestry are related by common ancestors in the recent past. Perhaps surprisingly, we do not have go too much further back before all humans share a common ancestor if we assume even little gene flow among major human geographical groups. The most recent common ancestor of all present‐day humans lived just a few thousand years ago (Rohde, Olson, & Chang, 2004).

## 5 PRACTICE

I try to remember that it's not me, John Seed, trying to protect the rainforest. Rather I'm part of the rainforest protecting myself. I am that part of the rainforest recently emerged into human thinking.

John Seed (in Macy, 1991: page 184)

The object of Zen practice is to develop acute awareness of the present moment. The cultivation of mindfulness is a Zen practice to develop sharpened awareness of the immediate present in which we strive to look deeply into our every action. This develops a deeper level of understanding so that we can act out of feeling or experience rather than out of intellectual knowledge. David Orr (1994) discusses the importance of “feeling” the truth in the final chapter of his book, *Earth in Mind*. He concludes that the objective of environmental education should be to draw out our affinity for life. We cannot act wisely without knowledge; we will not act wisely without feeling. Zazen is form of meditation used in Zen practice to center, focus, and quiet the mind. “Ordinarily the mind is so clouded with irrelevant thoughts, fantasies, worries, judgements, and desires that we are unable to see things as they truly are” (Graef, 1990).

### 5.1 Contemplation

Some people have experienced moments during meditation in which they felt a deep conviction of the interconnectedness of all life. Jane Goodall (2003: pages 173 and 175) has described her experience at Gombe Stream National Park: “Self was utterly absent: I and the chimpanzees, the earth and trees and air, seemed to merge, to become one with the spirit power of life itself…. In a flash of “outsight”, I had known timelessness and quiet ecstasy, sense a truth of which mainstream science is merely a small fraction.” Such awakenings can provide a basis for universal compassion and responsibility for the natural world. One can see how Goodall's intimate knowing of many individual chimpanzees and experiences such as this inspired and reinforced her dedication to conservation of chimpanzees and other wildlife.

The life story of Barbara McClintock (Keller, 1983) provides a fascinating parallel of such experiences leading to scientific understanding. McClintock gained incredible insight into the chromosomes of maize (*Zea mays*) by methods that she herself likened to the deep meditations of Tibetan Buddhists (Keller, 1983: page 202). She describes her own mental experience of being inside the cell to visualize how the chromosomes are behaving. I am struck by the similarity of Jane Goodall describing the behavior of the chimpanzees, which she came to know so well, with McClintock's description of the understanding the behavior of chromosomes. McClintock describes her difficulty of using the “so‐called scientific method,” not to understand the behavior of chromosomes, but rather in order to convince her colleagues of what she learned through deep contemplation (Keller, 1983: page 203).

### 5.2 Action

A number of practices have been developed by Zen practitioners that we can use to cultivate our feeling of affinity for all life. A deep understanding of our evolutionary relationships is recognized and reinforced in some Zen practices. For example, Thich Nhat Hanh (2004) has written the following passage to be contemplated as part of a ceremony called “Touching the Earth”: My spiritual ancestors and blood ancestors, my spiritual descendants and my blood descendants, are all part of me. I am them, and they are me. I do not have a separate self. All exist as part of a wonderful stream of life which is constantly moving.”

Gathas, short verses used to bring the energy of mindfulness to each act of daily life, are a traditional form of Zen practice used to increase our awareness. The following gatha, written by Thich Nhat Hanh (1992: page 104), can be used before every meal:

In this food

I see clearly the existence

of the entire universe,

supporting my existence.

We *can* see the entire universe in our breakfast cereal if we take just a moment to reflect. The ocean is there: the rain that watered the grain was carried from the ocean by clouds. The sun is there: the grain could not grow without energy from the sun. The Jurassic ecosystem in which the dinosaurs dwelled is there: plants that fed the dinosaurs, 200 million years ago were transformed into the fossil fuel that was used to harvest the grain and to carry it to the table. Gregor Mendel is there, along with the plant breeders who developed the strains of grain. Such moments of reflection strengthen our appreciation of our interdependence to countless beings, past and present, near and far.

Cultivating awareness of our actions is a powerful method to transform our behavior so that we can act in a way that will protect, maintain, and restore life on this planet. However, how we modify our behavior to protect life is an individual choice. Such awareness might inspire us to modify our daily habits (e.g., bike or walk to work rather than drive), or perhaps modify how we direct our scientific efforts. In my own experience, I have chosen to work on scientific problems that are potentially important for conservation (e.g., effects of hybridization and inbreeding), as well as being of basic scientific interest.

### 5.3 Salmon

We can develop daily practices that are appropriate for our own experiences. I have spent much of my life studying Pacific salmon (*Oncorhynchus* spp). Their anadromous life cycle, which includes residence in both freshwater and the ocean, makes it easy to see the connections among species and ecosystems. The return of salmon from the ocean is a crucial link in freshwater ecosystems throughout the North Pacific Rim. For example, up to 90% of the nitrogen in benthic algae from Sashin Creek in southeast Alaska is derived from the rotting carcasses of spawned‐out pink salmon (*Oncorhynchus gorbuscha*; Levy, 1997).

Sockeye salmon (*Oncorhynchus nerka*) from Redfish Lake of the Columbia River traverse many power‐generating dams during their journey to and from the ocean. We turn light switches on many times throughout our daily life without awareness. Mindfully performing this act requires awareness of the physical sensation of touching and moving the switch. In addition, we can become aware of the effects of this action. I live in a power grid connected to the power‐generating dams of the Columbia River. The connection I made when I turned on the light in my office this morning connected my office to electrical power generated by dams on the Columbia River. These dams and the long stagnant pools behind them have blocked or hindered the return of salmon to their spawning grounds. I try to be aware of that connection every time I turn on a light switch; I usually fail.

In addition, Pacific salmon have been an essential part of the diet for people living on the west coast of North America for thousands of years. We can see in the act of eating that the salmon literally become us. In the words of Mary Oliver (1983: page 56) from *The Fish*:

I opened his body and separated

the flesh from the bones

and ate him. Now the sea

is in me: I am the fish, the fish

glitters in me; we are

risen, tangled together, certain to fall

back to the sea.

## 6 CONCLUSION

Zen teaches that our identity is not limited to our ego‐self. Our identity includes all living beings. Humans act in a way that they feel will be in their own self‐interest. We will act to save “life on this planet” only if we recognize at a deep level that our “self” includes all beings. We need to recognize and feel at a deep level that ultimately we are not biologists trying to save other species. Rather, we are one emergence of life on this planet trying to save itself.

## CONFLICT OF INTEREST

None declared.
